# Routine Immunization Coverage and Immunization Card Retention in Pakistan: Results From a Cross-sectional National Survey

**DOI:** 10.1097/INF.0000000000003804

**Published:** 2023-01-02

**Authors:** Imtiaz Hussain, Ahmad Khan, Dale A. Rhoda, Imran Ahmed, Muhammad Umer, Uzair Ansari, Muhammad Akram Shah, Soofia Yunus, Jennifer Brustrom, Robert Oelrichs, Sajid Bashir Soofi, Zulfiqar A Bhutta

**Affiliations:** * Center of Excellence in Women & Child Health, The Aga Khan University, Pakistan; † Biostat Global Consulting, Worthington, OH; ‡ Federal Directorate of Immunization, Pakistan; § Independent Consultant, Brisbane, Australia; ¶ Department of Pediatrics & Child Health, The Aga Khan University, Pakistan.

**Keywords:** immunization coverage, Pakistan, national survey, vaccination, expanded program on immunization

## Abstract

Immunization is one of the most successful public health interventions available, saving millions of lives from death and disability each year. Therefore, improving immunization coverage is a high priority for the Government of Pakistan and essential to progress toward universal health coverage. This survey reports the national and provincial/regional coverage and determinants of fully, partially, and not-vaccinated children 12–23 months of age, antigen-wise coverage, percentage of home-based vaccination records (HBR) retention, and reasons for nonretention; dropout, timeliness, and prevalence of missed opportunities for simultaneous vaccination (MOSV).

**Methods:**

The survey was a descriptive cross-sectional national household survey carried out across Pakistan. The survey included 110,790 children 12–23 months old and their caregivers. A World Health Organization (WHO)—Expanded Program on Immunization (EPI) Survey questionnaire was adapted to collect information. Data were analyzed using the WHO Vaccination Coverage Quality Indicators (VCQI) software and Stata version 17.

**Results:**

Nationally excluding Azad Jammu and Kashmir (AJK) and Gilgit Baltistan (GB), the coverage of fully vaccinated children was 76.5%. The likelihood of being fully vaccinated was higher among children of educated parents who belonged to higher wealth quintiles and resided in any province/region other than Balochistan. The main reasons for unimmunization were no faith in immunization, rumors about vaccines, and distance to the facility. About two-thirds (66.2%) of the children had their HBR available, and the main reasons for not having a card were never visiting a health facility and having no awareness about the importance of a card. Dropout was discernible for later doses of vaccines compared with earlier ones. Higher proportions of children received the last doses late by more than two months. Of the 218,002 vaccination visits documented on HBR in the provinces, MOSVs occurred in 17.6% of the visits.

**Conclusion:**

The immunization coverage rates provide a direction to strategize the progress to improve the vaccination rates in Pakistan. The country needs to outline the immediate and long-term actions to combat vaccine-preventable diseases, such as escalating integrated immunization campaigns and outreach activities, provision of mobility support, and deploying behavioral interventions as a cross-cutting strategy to improve awareness and reduce misconceptions.

Immunization is one of the most successful public health interventions available, saving millions of lives from death and disability each year. Pakistan has the highest neonatal, infant, and child mortality globally,^1,2^ and most of these deaths are avoidable through simple public health interventions such as immunization. Moreover, Pakistan is one of two countries where polio is still endemic^3^ and ranks third in the world for having the highest number of unvaccinated children (1.2 million), after Nigeria (approximately 4 million) and India (2.9 million).^4^ Therefore, increasing immunization coverage is a high priority for the Government of Pakistan and an essential part of making progress toward universal health coverage and building resilient health systems.

Pakistan adopted the World Health Organization’s Expanded Program on Immunization (EPI) in 1976 on a pilot scale and expanded the program countrywide in 1978.^5^ The EPI in Pakistan covers ten vaccine-preventable diseases (VPDs) in their current form; however, despite significant efforts, Pakistan’s immunization indicators have yet to reach the expected benchmarks. The polio eradication and measles elimination goals have not been achieved^6^ as the country experiences continued incidents of endemic polio transmission and periodic measles outbreaks. Among the reasons for low immunization coverage, such as limited access to services, lack of awareness among communities, low socioeconomic status, and parental education, and gaps in vaccination service delivery, the EPI also faces inefficiency and unsustainability caused by the fragmented financial structure of the program and sub-optimal monitoring and evaluation system. Moreover, the devolution of power to the provinces established by the 18th Amendment to the Constitution in 2010 made the management of immunization and other health services the responsibility of the provinces, weakening already fragile healthcare delivery systems.^7^

In 2016, Pakistan started the National Immunization Support Project (NISP) with financial support from the World Bank, Gavi—the Vaccine Alliance, USAID, and the Bill and Melinda Gates Foundation (BMGF).^7,8^ NISP was launched to improve equitable coverage of immunization services against VPDs and further strengthen the existing EPI initiative through financial investment, programmatic reform, and efficiency improvement. In addition, to improve the immunization coverage across Pakistan, specifically in areas of low vaccine coverage, the NISP introduced a result-based approach in which funding was linked to province-level outcome targets defined in the form of Disbursement Linked Indicators (DLIs).^8^

Despite all these efforts, immunization in Pakistan has yet to realize its full potential, mainly because the success of an immunization program depends on high rates of acceptance and coverage in the population. It is, therefore, highly desirable to assess the coverage and achievements of immunization rates nationally to make future policy decisions more evidence-informed. Considering this, a Third-Party Verification Immunization Coverage Survey (TPVICS) was organized to assess the national and sub-national (province) rates and determinants of fully, partially, and not vaccinated children between 12 and 23 months old, along with reasons for unimmunization.

## METHODS

This survey was a descriptive cross-sectional national household survey carried out in all provinces in all provinces (Sindh, Punjab, Khyber Pakhtunkhwa (KP), Balochistan) and regions (federal regions; Islamabad, Azad Jammu and Kashmir (AJK), Gilgit-Baltistan (GB) and erstwhile Federally Administered Tribal Areas (FATA) now as Khyber Pakhtunkhwa-Newly Merged Districts (KP-NMD) of Pakistan between September 2020 and February 2021). The population of interest was children 12–23 months of age and their caregivers. Two project reports describe the survey methods in more detail.^9,10^

### Sample Size

The survey sample size was designed to estimate key vaccination indicators at the district level with moderate precision and province and national levels with high precision. The precision of a cluster survey outcome is impossible to predict accurately because the observed values of the parameters that affect it (estimated coverage, number of respondents per cluster, intracluster correlation coefficient, and coefficient of variation of the survey weights) are not known when the sample size calculation is conducted.^11^ Before the survey, administrative data were used to classify each district as either (1) likely to have 80% or more children fully vaccinated or (2) likely to have fewer than 80% of children fully vaccinated. Districts likely to have high coverage were allocated 49 primary sampling units (PSUs or clusters) each, and those likely to have lower coverage were allocated 64 clusters. In each survey cluster, 13 households containing eligible children were selected for interviews. If each cluster produced an average of ten completed interviews, and if the design effect turned out to be no higher than 2.5, then precision for the 49 cluster districts would be ±6% if 85% of children were fully vaccinated. In the 64 cluster districts, if the design effect was no larger than 2.5, precision would be ±6% if 75% of children were fully-vaccinated.^12^

### Sampling Strategy

The survey strata were the 152 health districts of Pakistan. Within each district, respondents were selected in two stages. First, the Pakistan Bureau of Statistics (PBS) selected enumeration areas as PSUs using the 2017 Pakistan Census sampling frame. Next, every household was visited in every survey PSU to screen for the presence of children 12–23 months of age. Households with eligible children were treated as the sampling frame for secondary sampling units. In every PSU, 13 households with eligible children were selected for the sample and visited for interviews. Data were collected on all eligible children in all the selected households.

### Data Collection and Analysis

A WHO survey questionnaire for EPI coverage was adapted to include the national immunization schedule and socio-demographic indicators to assess households, dwellings, and children 12–23 months old. The immunization schedule of Pakistan is presented in Table 1. The questionnaire was translated into the national language (Urdu) and pilot field-tested on 1,000 samples in 20 different nationwide locations. Standard operating procedures and manuals were developed and followed for household listing and data collection. The survey implementation team consisted of data collectors, data supervisors, and district and provincial supervisors hired and trained for five days in Karachi by investigators and faculty members of the Aga Khan University and facilitated by the WHO and EPI program authorities.

**TABLE 1.** Expanded Programme on Immunization Schedule for Children in Pakistan

When	Age	Vaccines
At birth	At birth	BCG, OPV0, HepB
2^nd^ visit	6 weeks	OPV1, Rotavirus1, PCV1, Pentavalent1
3^rd^ visit	10 weeks	OPV2, Rotavirus2, PCV2, Pentavalent2
4^th^ visit	14 weeks	OPV3, IPV1, PCV3, Pentavalent3
5^th^ visit	9 months	Measles1, IPV2*, typhoid*
6^th^ visit	15 months	Measles2

* The TPVICS survey did not ask about IPV2 or typhoid and neither were implemented country-wide at the time the children in this survey were 9 months old.

Data were collected in two stages. First, households were visited and screened for eligibility for selection; after thirteen households per PSU were selected, they were visited for the interview to collect evidence on routine immunization and information on household socioeconomic status. Vaccination evidence was preferred from an HBR. If the card was available, it was photographed, and the relevant dates were transcribed. In addition, the caregivers were asked to recollect which doses the child had received for children without cards. Android handheld devices were used for data collection. Data was transmitted to the AKU data center in real-time. Survey responses were checked for accuracy and completeness daily, and photographs of vaccination cards were rechecked on a set of randomly selected revisits by supervisors. Apparent inconsistencies in individual vaccination records were detected using a set of computerized logic checks to evaluate the vaccination dates transcribed from cards. When logically incompatible dates were detected (e.g., vaccination before the child was born; dose two given before dose one), field team supervisors and data quality personnel re-reviewed the cards’ digital photos and made appropriate database corrections.

The data were analyzed using the freely available WHO software, Vaccination Coverage Quality Indicators (VCQI)^13^ implemented in Stata version 17.^14^ VCQI estimated the vaccination coverage, considering evidence from cards or caregiver recall, including the percentage of children who were fully, partially, or not-vaccinated, dropout, and home-based vaccination record availability.

The following information was captured, if a vaccination card was available; timeliness of vaccination, compared with the target age for each dose as expressed in EPI–Pakistan’s recommended immunization schedule, interval (in days) between doses, and visits with MOSV.

A child was categorized as fully vaccinated when s/he completed vaccination from birth through the first dose of measles-containing-vaccine (MCV1), which is scheduled to be given at nine months of age, as per the schedule of the EPI,^15^ i.e., BCG, OPV0, OPV1-3, Penta1-3, PCV1-3, IPV, and MCV1. ROTA1 and ROTA2 are not included in full immunization because they are the doses introduced into the EPI schedule most recently. A child was categorized as partially vaccinated when s/he received at least one but missed at least one of those doses. A child was categorized as not vaccinated if s/he did not have evidence of receiving any of those doses. Data were not collected on IPV2 or typhoid vaccine because those doses had not been implemented nationally when the children in this survey were 9 months old.

PBS provided sampling weights at the cluster level to account for unequal selection probabilities and non-response. The weights were certified to aggregate results across districts to estimate coverage at the province, region, and national levels. The survey outcome variance calculations account for stratification, clustering, and weights and report the proportion of vaccination coverage with 95% survey-adjusted Wilson confidence intervals.^16^ Results were summarized in tables, and figures. Survey-adjusted logistic regression was used to explore factors correlated with full, partial, and no immunization. Initial regressions included a single independent variable at a time. Only variables with *P* < 0.25 in a bivariate model were retained in the multivariable model.

### Ethics

Ethical clearance was obtained by the National Bioethics Committee (NBC) No.4-87/NBC-379/19/1996 and the Aga Khan University (AKU) Ethical Review Committee (ERC) 2019-0652-5699. Before seeking the ethics clearance, a letter of support was obtained from the National Program Manager EPI, Ministry of National Health Services Regulation & Coordination (MoNHSRC) Islamabad, and the provincial authorities’ collaboration approvals with PBS.

## RESULTS

### Sample Description

Table 2 describes the sample. The survey covered 8,759 clusters and 109,123 households, including 110,790 children across the country, and the overall response rate was 96.5%. The national mean household size was 6.9 (SD ± 3.7), with the highest in GB (9.9 ± 5.2) and FATA (KP-NMD) (9.9 ± 6.2) and the lowest in Sindh (6.1 ± 3.0). The mean age of children was 17.6 months (SD ± 3.4), and 54% (*n* = 59,872) of children were male, and the proportion was the highest in Balochistan (57.7%) and FATA (KP-NMD) (56.6%). Most of the population belonged to the combination of affluent and wealthiest wealth quintiles (60.1), particularly in Punjab (70.4%) and AJK (63%). More than half of all mothers (52.1%) and fathers (62.6%) were literate; overall, literacy was highest in AJK.

**TABLE 2.** Basic Characteristics of Survey Sample

Level	Clusters			Households					Children 12–23 months			Wealth Quintiles					% literate*	
	Sampled	Randomized	Surveyed	Target	Randomized	Completed	Response Rate (%)	Household size (mean ± sd)	N	Age in months (mean ± sd)	% Male children	Poorest	Poor	Middle	Rich	Richest	Mothers	Fathers
Pakistan	8,786	8,759	8,759	114,218	113,057	109,123	96.5	6.9 ± 3.7	110,790	17.6 ± 3.4	54.04	10.2	13	17	23	36.9	52.1	62.6
KP	1,386	1,378	1,378	18,018	17,878	16,967	94.9	8.6 ± 5.1	17,432	17.8 ± 3.4	52.7	5	14	25.9	31	24	42.4	62.4
Punjab	1,839	1,839	1,839	23,907	23,898	23,763	99.4	6.7 ± 3.3	24,037	17.5 ± 3.4	52.2	3.6	9.4	16.7	26	44.7	62.1	69.3
Sindh	1,856	1,855	1,855	24,128	24,086	23,006	95.5	6.1 ± 3.0	23,290	17.4 ± 3.5	52.6	21.3	16	12.6	17	33.8	41.5	55.3
Balochistan	2,112	2,094	2,094	27,456	26,591	25,431	95.6	8.4 ± 5.2	25,764	18.0 ± 3.1	57.7	34.7	25	21.6	11	7.5	16.6	21.7
Islamabad	113	113	113	1,469	1,468	1,439	98	6.8 ± 3.0	1,458	17.8 ± 3.4	48.9	1.5	3.6	5.9	18	70.5	74.8	82.7
GB	433	433	433	5,629	5,628	5,390	95.8	9.9 ± 5.2	5,483	17.6 ± 3.5	52.7	12.6	32	28.3	18	9	55.5	70.9
AJK	580	580	580	7,540	7,540	7,462	99	7.4 ± 3.5	7,547	17.8 ± 3.3	52.6	3.4	11	22.7	42	21.5	79.9	88.6
FATA (KP-NMD)	467	467	467	6,071	5,968	5,665	94.9	9.9 ± 6.2	5,779	18.2 ± 3.3	56.6	28.4	37	23	9.8	2.2	16.9	30.6

AJK, Azad Jammu & Kashmir; GB, Gilgit Baltistan; KP, Khyber Pakhtunkhwa; FATA, Federally administered tribal areas; KP-NMD, Khyber Pakhtunkhwa’s newly merged districts.

* With one or more years of education.

### Immunization Cards

Table 3 summarizes HBR availability rates and the reasons for their non-availability. HBR availability across all provinces was 66.2%, highest in Punjab (80.8%) and lowest in Balochistan (19%). The proportion of children whose cards were shown to the surveyors was smaller than the proportion who received a vaccination card (87.1%), ranging from 94.7% in Punjab to a low of 48.4% in Balochistan. The main reasons for not showing immunization cards across Pakistan were no awareness of the importance of the card (verbalized by 3.2% of the population), never visiting a facility (2.6%), and nonavailability of the card at a health facility at the time of visit (1.5%). Other reasons highlighted that a card was not provided by a health care provider (1.3%) and was unaware of any such card (1.3%). The prevalence of these reasons was higher in Balochistan and FATA (KP-NMD).

**TABLE 3.** Card Availability and Reasons Children were Missing Cards (%)

Level	12–23 months children(*N*)	Ever had a card	Reasons the card was not available						Card Availability (seen at the time of interview)
			Don notthink it is important	Never Visited a facility	Card not available with the health provider	Vaccinator/ facility did not provide the card	Not aware of such card	Other	
Pakistan *	97,760	87.1	3.2	2.6	1.5	1.3	1.3	3.1	66.2
KP	17,432	80.9	5.3	5.5	1.7	1.6	2.0	3.1	57.3
Punjab	24,037	94.7	1.2	0.3	1.0	0.3	0.2	2.4	80.8
Sindh	23,290	83.3	3.6	2.8	1.8	2.8	2.0	3.7	50.0
Balochistan	25,764	48.4	14.0	16.3	3.7	3.2	8.1	6.3	19.0
Islamabad	1,458	83.5	1.7	0.8	4.2	0.1	2.2	7.5	61.7
GB	5,483	84.2	1.3	2.7	1.4	2.0	1.9	6.5	52.5
AJK	7,547	96.5	0.4	0.5	0.7	0.2	0.1	1.6	76.4
FATA (KP-NMD)	5,779	53.4	17.5	17.0	1.4	3.5	4.0	3.2	40.4

AJK, Azad Jammu & Kashmir; GB, Gilgit Baltistan; KP, Khyber Pakhtunkhwa; FATA, Federally Administered Tribal Areas; KP-NMD, Khyber Pakhtunkhwa’s newly merged districts.

* Excludes AJK and GB.

### Immunization Coverage

Table 4 shows the proportion of fully, partially, and not vaccinated children in each province and region based on immunization information collected from both vaccination cards and recall by mothers/caregivers of the targeted children. The results show that national coverage of being fully, partially, and not vaccinated was 76.5%, 18.1%, and 5.4%, respectively. The proportion of fully vaccinated was highest in Punjab (90%), followed by AJK (88.9%) and GB (73.4%), and lowest in Balochistan (37.7%). The proportion of not vaccinated (also known as zero-dose) children was highest in FATA (KP-NMD) (34.2%), followed by Balochistan (31.5%).

**TABLE 4.** Percent of Children 12–23 Months of Age who were Fully-, Partially-, and Not Vaccinated

Level	Fully vaccinated^§^(%)	Partially vaccinated(%)	Not vaccinated(%)
Pakistan*	76.5	18.1	5.4
KP	68.5	20.6	10.8
Punjab	90.0	9.5	0.6
Sindh	61.2	31.9	6.9
Balochistan	37.7	30.8	31.5
Islamabad	71.0	26.1	2.9
GB	73.4	22.7	3.9
AJK	88.9	10.2	0.9
FATA (KP-NMD)	42.9	22.8	34.2

Fully vaccinated means that the child received BCG, OPV0, OPV1-3, PENTA1-3, PCV1-3, IPV, and MCV1. Coverages are based on both vaccination cards and recall by mothers/caregivers.

* Excludes AJK and GB.

### Antigen-Wise Vaccination Coverage

**FIGURE 1.**
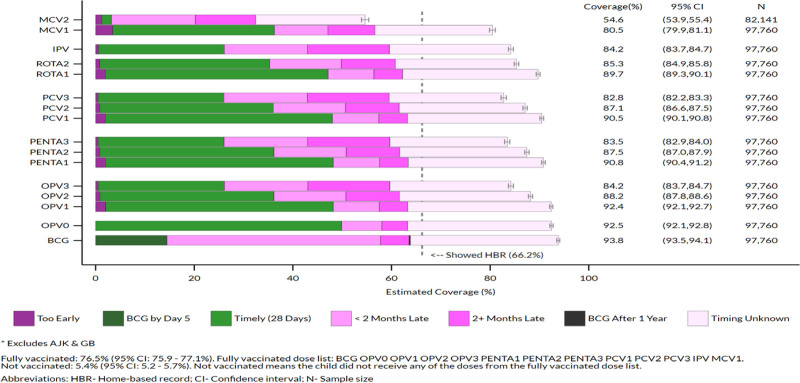
Vaccination coverage and timeliness for children 12–23 months of age, Pakistan*.

Table 5 presents antigen-wise coverage estimates, and Figure 1 illustrates coverage graphically for the combined provinces of Pakistan (excluding AJK and GB) and summarizes the timeliness of each dose using several categories. According to the national immunization schedule (Table 1), timeliness is the difference between the age when the child received the dose and the age when they are eligible for that dose. The online folder^17^ that supplements the VCQI analysis results report^10^ holds similar coverage and timeliness charts for each province, region, and district.

**TABLE 5.** Antigen-Wise Coverage Estimates (%)

	Birth		6 weeks				10 weeks				14 weeks				9 months
	BCG	OPV0	OPV1	PENTA1	PCV1	RV1	OPV2	PENTA2	PCV2	RV2	OPV3	PENTA3	PCV3	IPV	MCV1
Pakistan*	93.8	92.5	92.4	90.8	90.5	89.7	88.2	87.5	87.1	85.3	84.2	83.5	82.8	84.2	80.5
Provinces and regions															
Punjab	99.2	98.5	98.6	98.3	98.2	97.6	97.3	97.1	96.8	95.6	95.0	94.9	94.5	95.4	92.6
Sindh	92.5	90.9	89.1	86.3	85.8	84.6	81.4	79.7	79.2	76.2	75.0	73.2	71.5	73.7	67.2
Khyber Pakhtunkhwa	88.0	85.9	86.7	84.6	83.9	83.2	80.5	79.8	79.3	77.7	75.3	74.3	73.7	75.9	73.1
FATA (KP-NMD)	63.5	58.3	61.6	59.8	59.0	59.2	55.1	54.0	53.7	53.4	51.2	49.6	49.5	51.3	49.5
Balochistan	63.1	58.0	63.1	54.2	53.5	53.0	51.0	47.7	47.4	46.4	45.3	42.2	42.0	44.7	42.7
Islamabad	96.9	96.0	95.1	94.5	94.1	93.1	85.9	92.5	92.1	90.8	80.4	86.3	86.2	88.1	81.8
AJK	98.9	98.8	98.9	98.6	98.6	98.2	97.6	97.9	97.9	97.4	93.0	95.4	95.6	95.9	93.2
GB	93.6	93.8	95.0	90.0	89.4	85.9	90.2	86.8	86.4	81.7	84.8	82.2	81.1	83.7	81.2

AJK, Azad Jammu & Kashmir; BCG, Bacille Calmette-Guérin; GB, Gilgit Baltistan; IPV, Inactivated Polio Vaccine; KP: Khyber Pakhtunkhwa; FATA, Federally Administered Tribal Authority; KP-NMD, Khyber Pakhtunkhwa’s newly merged districts; MCV, Meningococcal Conjugate Vaccine; OPV, Oral Polio Vaccine; PCV, Pneumococcal Conjugate Vaccine; Penta, Pentavalent Vaccine; RV: Rota Virus.

* Excludes AJK and GB.

Nationally, the immunization coverage for BCG was 93.8%, for OPV-0 it was 92.5%, OPV-1 92.4%, OPV-2 88.2%, and for OPV-3 it was 84.2%. For Penta doses, the national rates were recorded at 90.8% for Penta-1, 87.5% for Penta-2, and 83.5%. For Penta-3, whereas for PCV-1, PCV-2, and PCV-3, the rates were recorded at 90.5%, 87.1%, and 82.8%, respectively. For RV-1 and RV-2, the national coverage rates were 89.7% and 85.3%, respectively, whereas for IPV the coverage was 84.2%, and for MCV-1 it was 80.5%. For all these vaccines, the coverage rate was highest in Punjab and AJK and lowest in Balochistan and FATA (KP-NMD).

### Dropout

Ideally, no child would drop out of the immunization system, and all would receive all the doses in the national immunization schedule. Dropout is visually discernible in comparing the shorter length of coverage bars in Figure 1 for later doses with earlier ones. The supplements’ outcomes for several dose pairs are available in the full VCQI analysis report.^17^

### Indicators that Rely upon Dates

#### Vaccination Timeliness

The dark pink bar segments near the center of Figure 1 represent children who received the dose two or more (2+) months after the scheduled age. For doses that fall later in the immunization schedule, higher proportions of children received the dose 2+ months late. Table 6 expresses the portion who received the dose 2+ months late as a proportion of those who received the dose and for whom timing is known. More children received the last doses 2+ months late than earlier in every province and region. For example, in FATA (KP-NMD), 40.4% of children showed cards (Table 3), and among children whose dose age was known for doses due at 14 weeks, two-thirds (68.4–68.8%) received the dose 2+ months late (Table 6). All timeliness categories are visually apparent in the online folder’s coverage and timeliness charts, which supplement the VCQI analyses report.^10,17^

**TABLE 6.** Percent of Children Who Received the Dose Two or More Months Late, Among Those for Whom Dose Timing is Known (%)

Due at	Dose	Punjab	Sindh	KP	FATA (KP-NMDs)	Balochistan	Islamabad	AJK	GB
6 weeks	OPV1	5.9	13.9	18.3	33.6	31.4	5.6	2.9	11.2
	PENTA1	5.8	13.9	18.5	33.6	32.9	5.5	2.9	11.1
	PCV1	5.9	14.0	18.5	33.5	33.1	5.7	2.8	11.3
	ROTA1	5.9	14.7	18.8	33.9	32.9	5.9	2.8	11.1
10 weeks	OPV2	12.4	25.0	35.9	52.0	38.3	13.0	6.7	20.0
	PENTA2	12.3	25.1	36.0	51.9	39.7	13.2	6.7	20.1
	PCV2	12.3	25.5	36.0	51.9	40.4	13.2	6.7	20.1
	ROTA2	12.6	26.3	36.5	52.8	40.4	13.4	6.6	20.3
14 weeks	OPV3	21.6	36.6	54.0	68.8	52.0	23.8	14.6	29.9
	PENTA3	21.7	36.4	54.1	68.5	52.8	24.1	14.6	29.8
	PCV3	21.5	36.6	54.4	68.4	52.3	24.4	14.6	30.1
	IPV	21.4	37.3	54.6	68.4	52.7	24.1	14.6	29.9
9 months	MCV1	12.2	27.0	31.3	41.9	36.6	17.8	11.1	20.0
14 months	MCV2	35.5	46.2	41.9	51.6	40.9	52.3	37.6	40.4

The denominator excludes children who did not receive the dose and children for whom the dose timing (age) is unknown.

#### Missed Opportunities for Simultaneous Vaccination (MOSVs)

A missed opportunity for simultaneous vaccination (MOSV) occurs when a child has contact with the health system and receives at least one vaccine dose but does not receive all the doses for which s/he is eligible.^18,19^ Findings from our survey reflected that MOSVs occurred in 17.6% of vaccination visits across the provinces.^10^ Of the 218,002 vaccination visits documented on home-based vaccination records in the provinces, the child did not have a MOSV on 82.4% of visits. That is to say that the child received all the doses s/he was eligible for on 179,634 of those visits. Performance in AJK and GB was slightly better, with MOSVs on only 7.3% and 15.7% of visits there, respectively.

Table 7 documents MOSVs by dose among children. A high proportion of children who experienced MOSVs for IPV (49.3%) across provinces varies 25.5% in AJK and 75.1% in FATA (KP-NMD)) and the notable proportion who experienced MOSVs for MCV1 (7.9%) across the provinces ranging from 1.5% in Punjab to 26.1% in FATA (KP-NMD). The IPV MOSVs occurred when the child came late to receive the doses scheduled to be given at ten weeks or even six weeks. If the child had reached the age of 14 weeks when receiving the doses due at six or ten weeks, they were also eligible to receive IPV, but in most cases, did not receive it. Similarly, the MOSVs for MCV1 occurred when children came very late for doses scheduled for ten or fourteen weeks. If the child was already nine months old when they received the fourteen-week doses, they could have also been given MCV1, but in these instances, they were not. At the time of the survey, 12.8% of the children with IPV MOSVs had not yet received IPV across the provinces. In MOSV parlance, we say that the MOSV was uncorrected. Similarly, 40.5% of the MCV1 MOSVs had not been corrected. At the time of the survey, children with uncorrected MOSVs were still un- or under-protected against polio and measles, respectively.

**TABLE 7.** Percent of Children with One or More Missed Opportunities for Simultaneous Vaccination (%)

Due at	Dose	Punjab	Sindh	KP	FATA (KP-NMD)	Balochistan	Islamabad	Pakistan*	AJK	GB
Birth	BCG	17.8	3.8	4.7	14.6	6.8	1.3	10.9	0.6	1.0
	OPV0	1.7	1.3	0.7	2.5	2.1	0.6	1.4	0.0	0.7
6 weeks	OPV1	3.8	6.4	7.5	11.2	13.8	4.9	6.1	5.2	10.5
	PENTA1	3.8	6.3	7.5	11.1	14.0	5.0	6.1	5.2	10.4
	PCV1	3.8	6.7	7.6	11.1	13.8	4.9	6.2	5.2	10.3
	ROTA1	4.1	7.7	7.8	11.6	14.9	5.6	6.7	5.7	11.3
10 weeks	OPV2	0.4	1.0	0.9	2.6	2.5	0.5	0.8	0.2	0.8
	PENTA2	0.6	1.0	0.9	2.6	2.5	0.5	1.0	0.2	0.8
	PCV2	0.4	1.0	0.9	2.7	3.2	0.9	0.9	0.1	0.6
	ROTA2	0.7	1.6	1.2	3.5	3.7	1.3	1.4	0.2	1.0
14 weeks	OPV3	0.7	1.6	1.3	1.8	3.6	1.0	1.2	0.1	0.8
	PENTA3	1.3	1.6	1.3	1.9	3.1	1.0	1.5	0.1	0.7
	PCV3	0.8	1.6	1.3	2.2	3.3	1.3	1.3	0.1	0.7
	IPV	36.9	51.7	64.1	75.1	66.8	40.7	49.3	25.5	46.0
9 months	MCV1	1.5	11.7	12.3	26.1	23.1	6.5	7.9	2.1	5.5
14 months	MCV2	0.2	0.5	0.6	3.4	2.7	0.7	0.5	0.0	0.2
*N* **		20,045	10,618	8,967	2,605	3,167	904	46,306	5,786	2,952

* Excludes AJK & GB.

** *N* with card and with at least one vaccination date when they were eligible for one or more doses.

Also notable in Table 7 are the 10.9% of children who experienced MOSVs for BCG. These mostly occurred when the child received OPV0 some days before receiving BCG, perhaps because of a practice of only opening the 20-dose BCG vials when there are enough children present. In Pakistan (excluding AJK & GB), 89.4% of MOSVs for BCG were corrected, meaning that the child received the dose sometime after their first eligible vaccination visit. In Punjab and FATA (KP-NMD), the areas with the highest percent of children with MOSVs for BCG, the median time to correction was 9 days and 35 days, respectively. In Punjab, the children came back shortly after having received OPV0 to be given BCG on a day when a vial was open and long before they were due for their 6-week doses. In FATA (KP-NMD), the BCG missed opportunity was often corrected at a later vaccination visit when other doses were due.

#### Reasons for Nonimmunization

The reasons for not immunizing a child are presented in Table 8. The primary reasons for not vaccinating children are no faith in immunization (1.6%), rumors about getting vaccination (1.5%), and distance to the facility (1%).

**TABLE 8.** Reasons for not Immunizing Children (%)

Reasons	Pakistan*	KP	Punjab	Sindh	Balochistan	Islamabad	GB	AJK	FATA (KP-NMD)
Place of immunization too far	1.0	1.7	0.1	1.4	6.5	0.0	0.5	0.2	8.4
Time of immunization not convenient	0.2	0.4	0.0	0.2	1.3	0.0	0.0	0.0	1.9
Mother too busy	0.5	0.7	0.1	0.7	2.9	0.0	0.1	0.1	0.6
Family problem including mother ill	0.3	1.1	0.0	0.4	1.5	0.1	0.2	0.1	1.9
Child ill, not brought	0.4	1.0	0.0	0.7	1.8	0.1	0.2	0.1	1.5
Child ill, brought but not vaccinated	0.1	0.2	0.0	0.2	0.7	0.0	0.0	0.0	0.2
Long wait	0.1	0.2	0.0	0.2	0.9	0.0	0.1	0.1	0.2
Rumors	1.5	3.9	0.2	1.6	8.6	0.4	0.5	0.0	11.2
No faith in immunization	1.6	3.6	0.2	2.6	5.2	1.8	1.3	0.3	10.1
Fear of side reaction	0.6	1.1	0.1	1.0	3.1	0.0	0.2	0.1	2.8
Time or Place of immunization not Known	0.4	0.5	0.0	0.4	3.6	0.2	0.1	0.0	1.3
Took child but no vaccine available	0.0	0.0	0.0	0.1	0.3	0.1	0.1	0.0	0.1
Took child but no vaccinator	0.0	0.1	0.0	0.0	0.5	0.1	0.0	0.0	0.2
Took child facility closed	0.0	0.1	0.0	0.0	0.3	0.0	0.0	0.0	0.2
Child was sick	0.3	0.5	0.0	0.3	2.6	0.0	0.2	0.1	0.4
Took child but not vaccination day	0.0	0.1	0.0	0.0	0.3	0.0	0.0	0.0	0.1
Other	0.5	0.7	0.2	0.9	2.1	0.5	0.9	0.1	0.4

AJK, Azad Jammu & Kashmir; GB, Gilgit Baltistan; KP, Khyber Pakhtunkhwa; FATA, Federally Administered Tribal Areas; KP-NMD, Khyber Pakhtunkhwa’s newly merged districts

* Excludes AJK & GB.

#### Logistic Regression

Factors associated with being fully, partially, and not vaccinated children are presented in Table 9. The multivariable logistic regression indicates that the sex of the child was not associated with the immunization status. However, the rates of partially vaccinated children were lower among those residing in urban settings [OR 1.52; 95% CI, 1.37–1.70] than those living in rural settings. The place of residence was not associated with being fully vaccinated or not vaccinated. Maternal education and paternal education, respectively, were associated with being fully vaccinated. The full immunization rate was significantly higher among children of educated mothers and fathers. The likelihood of being fully vaccinated increased with increasing parental education (maternal secondary education [OR 1.34; 95% CI, 1.21–1.49], and higher education, i.e., 11 years and above [OR 1.66; 95% CI, 1.46–1.90]); paternal secondary education [OR 1.32; 95% CI, 1.20–1.44], and higher education, i.e., 11 years and above [OR 1.51; 95% CI, 1.35–1.68]. On the other hand, the rates of being partially vaccinated or not vaccinated were lower among children of educated mothers and fathers.

**TABLE 9.** Correlates of Full Immunization, Partial Immunization, and no Immunization

	Full immunization				Partial Immunization				No immunization			
	Univariate		Multivariable		Univariate		Multivariable		Univariate		Multivariable	
	OR (95% CI)	*P* value	OR (95% CI)	*P* value	OR (95% CI)	*P* value	OR (95% CI)	*P* value	OR (95% CI)	*P* value	OR (95% CI)	*P* value
Sex of child												
Male	Ref.				Ref.				Ref.			
Female	1.02 (0.98–1.07)	0.338			0.99 (0.95–1.04)	0.722			0.95 (0.89–1.01)	0.117		
Residence												
Rural	Ref.				Ref.		Ref.		Ref.			
Urban	1.02 (0.93–1.11)	0.719			1.29 (1.17–1.41)	0.000	**1.52 (1.37–1.70**)	0.000	0.39 (0.34–0.46)	0.000		
Maternal years of education												
None	Ref.		Ref.		Ref.		Ref.		Ref.		Ref.	
Primary (1–5)	2.37 (2.15–2.61)	0.000	**1.22 (1.10–1.35**)	0.000	0.58 (0.52–0.64)	0.000	**0.87 (0.78–0.97**)	0.011	0.21 (0.18–0.26)	0.000	**0.66 (0.55–0.80**)	0.000
Middle (6–8)	2.09 (1.86–2.35)	0.000	1.05 (0.93–1.19)	0.416	0.66 (0.58–0.75)	0.000	0.99 (0.87–1.13)	0.935	0.23 (0.19–0.27)	0.000	**0.70 (0.56–0.87**)	0.001
Secondary (9-10)	2.50 (2.28–2.74)	0.000	**1.34 (1.21–1.49**)	0.000	0.58 (0.53–0.64)	0.000	**0.80 (0.72–0.89**)	0.000	0.14 (0.11–0.16)	0.000	**0.46 (0.38–0.55**)	0.000
Higher (11 and above)	3.22 (2.86–3.63)	0.000	**1.66 (1.46–1.90**)	0.000	0.47 (0.41–0.53)	0.000	**0.64 (0.56–0.73**)	0.000	0.07 (0.06–0.09)	0.000	**0.31 (0.24–0.40**)	0.000
Paternal years of education												
None	Ref.		Ref.		Ref.		Ref.		Ref.		Ref.	
Primary (1-5)	1.85 (1.68–2.04)	0.000	1.05 (0.94–1.16)	0.390	0.71 (0.64–0.78)	0.000	0.98 (0.89–1.09)	0.749	0.33 (0.28–0.38)	0.000	0.94 (0.80–1.11)	0.481
Middle (6-8)	2.40 (2.17–2.66)	0.000	**1.23 (1.09–1.38**)	0.001	0.55 (0.50–0.63)	0.000	**0.86 (0.76–0.97**)	0.013	0.25 (0.22–0.29)	0.000	**0.80 (0.66–0.94**)	0.010
Secondary (9-10)	2.37 (2.19–2.56)	0.000	**1.32 (1.20–1.44**)	0.000	0.57 (0.53–0.63)	0.000	**0.80 (0.73–0.88**)	0.000	0.24 (0.21–0.27)	0.000	**0.83 (0.71–0.98**)	0.026
Higher (11 and above)	2.56 (2.33–2.81)	0.000	**1.51 (1.35–1.68**)	0.000	0.57 (0.52–0.63)	0.000	**0.74 (0.66–0.82**)	0.000	0.15 (0.13–0.18)	0.000	**0.63 (0.53–0.76**)	0.000
Wealth quintiles												
Poorest	Ref.		Ref.		Ref.		Ref.		Ref.		Ref.	
Poor	1.64 (1.48–1.81)	0.000	1.10 (1.00–1.22)	0.056	0.77 (0.70–0.84)	0.000	1.03 (0.94–1.14)	0.541	0.57 (0.51–0.65)	0.000	**0.77 (0.67–0.88**)	0.000
Middle	2.68 (2.42–2.96)	0.000	**1.34 (1.21–1.49**)	0.000	0.56 (0.50–0.62)	0.000	0.91 (0.82–1.02)	0.094	0.30 (0.26–0.35)	0.000	**0.54 (0.47–0.62**)	0.000
Rich	3.79 (3.39–4.23)	0.000	**1.46 (1.30–1.64**)	0.000	0.46 (0.42–0.52)	0.000	**0.83 (0.74–0.93**)	0.001	0.14 (0.12–0.16)	0.000	**0.39 (0.33–0.46**)	0.000
Richest	4.48 (4.03–4.99)	0.000	**1.30 (1.16–1.47**)	0.000	0.44 (0.40–0.49)	0.000	**0.83 (0.72–0.95**)	0.007	0.06 (0.05–0.07)	0.000	**0.30 (0.24–0.37**)	0.000
Province												
BALOCHISTAN	Ref.		Ref.		Ref.		Ref.		Ref.		Ref.	
KHYBER PAKHTUNKHWA	3.59 (3.18–4.06)	0.000	**2.72 (2.40–3.08**)	0.000	0.59 (0.51–0.67)	0.000	**0.75 (0.65–0.86**)	0.000	0.26 (0.23–0.30)	0.000	**0.46 (0.40–0.53**)	0.000
PUNJAB	14.82 (12.96–16.96)	0.000	**10.82 (9.43–12.41**)	0.000	0.23 (0.20–0.27)	0.000	**0.29 (0.25–0.34**)	0.000	0.01 (0.01–0.02)	0.000	**0.03 (0.02–0.04**)	0.000
SINDH	2.61 (2.33–2.92)	0.000	**2.12 (1.89–2.38**)	0.000	1.05 (0.93–1.19)	0.402	**1.15 (1.01–1.31**)	0.038	0.16 (0.14–0.19)	0.000	**0.22 (0.19–0.26**)	0.000
ISLAMABAD	4.04 (3.10–5.25)	0.000	**2.35 (1.72–3.23**)	0.000	0.79 (0.57–1.11)	0.178	1.16 (0.82–1.63)	0.400	0.07 (0.04–0.11)	0.697	**0.22 (0.14–0.35**)	0.000
GILGIT-BALTISTAN	4.55 (3.89–5.31)	0.000	**3.36 (2.87–3.93**)	0.000	0.66 (0.56–0.78)	0.000	0.87 (0.73–1.03)	0.106	0.09 (0.07–0.12)	0.000	**0.13 (0.10–0.18**)	0.000
AZAD JAMMU & KASHMIR	13.18 (11.18–15.55)	0.000	**8.46 (7.12–10.06**)	0.000	0.26 (0.21–0.31)	0.000	**0.37 (0.31–0.45**)	0.000	0.02 (0.05–0.11)	0.000	**0.09 (0.03–0.07**)	0.000
FATA (KP- NMD)	1.24 (1.03–1.50)	0.023	**1.26 (1.03–1.51**)	0.021	0.67 (0.54–0.83)	0.000	**0.73 (0.58–0.91**)	0.005	1.13 (0.92–1.39)	0.000	1.12 (0.90–1.39)	0.310

Included children from AJK and GB.

Multivariable outcomes with *p* values smaller than 0.05 appear in bold font.

AJK, Azad Jammu & Kashmir; GB, Gilgit Baltistan; KP, Khyber Pakhtunkhwa; FATA, Federally Administered Tribal Areas; KP-NMD, Khyber Pakhtunkhwa’s newly merged districts; OR, odds ratio.

Compared to children who belonged to the poorest wealth quintile, the likelihood of being fully vaccinated was higher among children from poor, middle, rich, and very rich wealth quintiles; however, the rates of being partially vaccinated were lower among rich and very rich wealth quintile, and not-vaccinated was lower with increasing wealth quintile. Compared to children from Balochistan, the likelihood of being fully vaccinated was significantly higher, and unimmunized was substantially lower in all other provinces and regions; however, partial immunization was considerably lower in all provinces and regions, except for Islamabad.

## DISCUSSION

The past two years of COVID-19 have impacted all aspects of life and disrupted all essential health services. Most children in the sample for this survey were younger than ten months when COVID-19 struck, so they may have had some routine immunization services disrupted or delayed. Furthermore, not all children who had reached the age of nine or ten months would have been fully vaccinated, and those who tried to obtain their remaining doses may also have been delayed or possibly prevented by the disruptions.

Routine immunization services are the hardest hit by the suspension of mass vaccination campaigns in low- and middle-income countries.^20^ However, despite the pandemic and its associated widespread lockdowns, curfews, and social distancing, this national survey between Sep 2020 and Feb 2021 identified that three-fourths (76.5%) of the children between the ages of 12-23 months are fully vaccinated, while 18.1% were partially vaccinated and 5.4% were not vaccinated. Compared with the national immunization coverage rate reported in the 2018 Pakistan Demographic and Health Survey (PDHS), i.e., 65.6%, the current rate shows an almost 10% increase.^21^ Our survey also found that the rate of full immunization coverage was the highest in the province of Punjab (90%) and the lowest in Balochistan (37.7%), which is similar to the findings in 2018 PDHS, i.e., 80.3% in Punjab and 40% in Balochistan (40%).^21^ While the rate of partial immunization was 18.1% which was lower than the rates reported in PDHS 30%,^21^ the rates of unvaccinated children were almost similar (our survey 5.4%; PDHS: 4%). Trends were similar across provinces/regions in both surveys. The national and provincial Penta3 coverage in our survey was higher than reported in the last 2018 PDHS survey^21^; however, the ranking in coverage was similar, i.e., highest in Punjab and lowest in Balochistan. Similar rankings were found for other vaccines.

Earlier studies have reported that socioeconomic factors such as parental education,^22–24^ place of residence,^22,24,25^ wealth index,^24^ sex of a child, and the number of children in the household,^24,25^ maternal antenatal care utilization,^22–24^ understanding of child health and VPDs,^23–25^ and presence of vaccination card^22^ are associated with complete immunization coverage. We found a positive relationship between immunization coverage and parental education, and it was observed that vaccination coverage improved with an increased level of education. Like the findings from the earlier research, vaccination coverage was positively associated with wealth quintiles; the higher the wealth quintile, the better immunization coverage. However, compared with Balochistan, the vaccination rates were higher in all the provinces. However, data from Sindh indicated that in catch-up activities for missed immunization, parents were disproportionately vaccinating more boys than girls and more children in urban areas than rural areas.^26^ Our results suggested no difference in vaccination rates based on the sex of a child, and place of residence (urban/rural and provincial levels as well).

Of the children, who were not vaccinated, the main reasons identified were poor access to the vaccination site, rumors around vaccination, and no faith in immunization. However, these reasons were present in a very small percentage (less than 2%). Therefore, it is essential to take a comprehensive approach, i.e., improving demand and supply-side interventions to accelerate vaccination rates.

A vaccination card is a source of information and a reminder for caregivers. It is an effective tool for recording vaccines a child receives to track later. Therefore, our survey took photographic documentation to confirm the presence of immunization cards at the time. Our survey identified that 66.2% of the children had their cards, similar to that reported in PDHS (63% of cards were physically seen at the time of the interview).^21^ Our survey found that card retention was the highest in Punjab and the lowest in Balochistan. Comparing the findings with the PDHS, TPVICS reported higher rates of vaccination card retention for all provinces except for Balochistan (TPVICS 19%; PDHS: 21.4%).

The rate of immunization card retention has always been reported as low in LMICs. An earlier systematic review reported a varied range of card retention rates, i.e., a lowest of 20.7% in the Democratic Republic of Congo and a highest of 69.2% in South Africa.^27^ However, the retention has been similar in Afghanistan, i.e., 66%.^28^ Earlier studies reported several reasons for not retaining an immunization card, and that includes large household size and the number of children in the household,^29^ gender of the child,^29^ place of residence,^30^ caregiver education,^30^ and access to mass media.^29^ In contrast, antenatal care and facility birth utilization are associated with improved retention of vaccination cards.^31^ Our survey underscored user-end behavioral and supply-end unavailability factors with the retention of cards. Caretakers have either never attended the facility or attended but were never provided with such cards, or they lacked awareness of its importance when provided. It is, therefore, imperative to highlight the importance of immunization cards and their uses in terms of a physical reminder for a timely vaccine dose.^27^

The dates on the cards give insight into the timeliness of vaccination and whether the children received all the doses, they were eligible for on each visit. The assessment of missed opportunities yields the welcome news that children received all the doses they were eligible for on more than 85% of documented vaccination visits. However, the cards also indicate that a notable portion of doses for which the child’s age could be calculated was given more than two months late, and the portion increased for doses later in the schedule. For example, some children received doses due at ten or 14 weeks after the age of nine months and experienced missed opportunities to acquire the measles vaccine during the same visit. Similarly, across the provinces, half the children (49.3%) who had vaccination visits after the age of fourteen weeks experienced one or more MOSVs for IPV. This occurred primarily when they received the 6-week and 10-week doses after 14 weeks. This incidence of very late doses and MOSVs for IPV and MCV1 occurred both before and after the onset of COVID-19. Efforts to diminish dropout and improve timeliness—to bring children back to the health facility for their later doses in a timely manner—would improve the protection of children of Pakistan against vaccine-preventable diseases.

## STRENGTHS AND LIMITATIONS

The main strength of this survey was physically accessing/photographing the immunization card and collecting information from the card itself rather than relying on information from the caregiver alone or in combination with the card. In addition, TPVICS is the first-ever large-scale immunization-specific survey conducted at the district level to share reflective results of program coverage in the country. Despite the pandemic’s challenges, the surveyors’ dedication contributed to acquiring some quality data. However, notwithstanding the findings, there were some limitations in the survey. For example, some of the clusters in Sindh (01 cluster), KP (8 clusters), and Balochistan (18 clusters) were dropped from the scope of the survey owing to inaccessibility and security reasons.

## CONCLUSION

The findings from this survey inform Pakistan’s immunization coverage, which is essential to direct future strategies to progress the rates. Our findings reflected that the MOSVs for BCG are more prevalent in Punjab than elsewhere in the country. One reason the rates are higher in Punjab is that their OPV0 coverage is notably higher than FATA (KP-NMD) and Balochistan. More children receiving OPV0 means more occasions on which there can be a missed opportunity for BCG. This will also mean that the 20-dose BCG vials have greater opportunities for being opened and used.

While disruptions caused by the COVID-19 pandemic had a devastating impact on routine vaccination campaigns, supplementary immunization activities (SIA) arrangements are a way to go. With polio still endemic, Pakistan must prioritize immediate and long-term strategies to improve immunization rates. Actions are required for low-coverage districts by intensifying immunization operations and expanding outreach activities to uncovered areas. The long-term actions should include behavioral change interventions to emphasize the importance of vaccination and the retention of vaccination cards for proper documentation from user and research perspectives.

## ADDITIONAL NOTE

This research paper is developed based on the Third-Party Verification Immunization Coverage Survey (TPVICS 2020-21) Report prepared by the Centre of Excellence in Women and Child Health, The Aga Khan University, available at:

https://www.aku.edu/coe-wch/Documents/TPVICS%20Survey%20Report.pdf

and

The Third-Party Verification Immunization Coverage Survey (TPVICS 2020-21) Vaccination Coverage Quality Indicators (VCQI) Analyses Survey Report, February 2022, jointly prepared by the Centre of Excellence in Women and Child Health, The Aga Khan University, and Biostat Global Consulting. Available at http://www.biostatglobal.com/downloads/TPVICS_2020_VCQI_Report.pdf

## ACKNOWLEDGMENTS

We thank Aga Khan University, Pakistan, for supporting this survey. Furthermore, we are immensely grateful to all participants for their cooperation during the survey.

## AUTHOR CONTRIBUTIONS

SBS conceptualized, and designed the survey, provided senior supervision, assisted with interpreting the results, and reviewed the manuscript. IH developed the sampling strategy, survey tools and supervised the field activities. IH and AK drafted the manuscript. DAR performed the statistical analysis. IA and UA managed the data and performed the statistical analysis. MU assisted with study design, formulating contextually relevant survey tools, and oversaw data collection. ZAB, MA, SY, JB, and RO critically reviewed the manuscript. All authors reviewed and approved the final manuscript for submission.
